# 4-[(3,4-Dimeth­oxy­benzyl­idene)amino]-1,5-dimethyl-2-phenyl-1*H*-pyrazol-3(2*H*)-one

**DOI:** 10.1107/S1600536810023238

**Published:** 2010-06-23

**Authors:** Abdullah M. Asiri, Salman A. Khan, Kong Wai Tan, Seik Weng Ng

**Affiliations:** a Chemistry Department, Faculty of Science, King Abdul Aziz University, PO Box 80203, Jeddah 21589, Saudi Arabia; b Department of Chemistry, University of Malaya, 50603 Kuala Lumpur, Malaysia

## Abstract

The imino–carbon double-bond in the title Schiff base, C_20_H_21_N_3_O_3_, has an *E* configuration; the six-membered aromatic substituent (r.m.s. deviation = 0.012 Å) is nearly coplanar with five-membered pyrazole substituent (r.m.s. deviation = 0.031 Å), the dihedral angle between the two systems being 11.4 (1)°]. The phenyl ring connected to the pyrazole ring is aligned at 45.5 (1)° with respect to this five-membered ring. The N atoms in the ring show pyramidal coordinations.

## Related literature

For background literature on Schiff bases derived from 4-amino­anti­pyridine, see: Montalvo-González & Ariza-Castolo (2003 ▶).
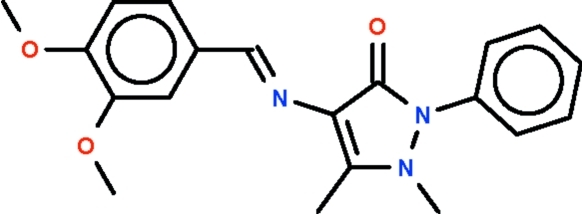


## Experimental

### 

#### Crystal data

C_20_H_21_N_3_O_3_
*M* *_r_* = 351.40
Monoclinic, 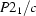

*a* = 12.5584 (8) Å
*b* = 10.4752 (7) Å
*c* = 14.6002 (9) Å
β = 109.039 (1)°
*V* = 1815.6 (2) Å^3^
*Z* = 4
Mo *K*α radiation
μ = 0.09 mm^−1^
*T* = 100 K
0.35 × 0.25 × 0.15 mm

#### Data collection

Bruker SMART APEX diffractometer
16900 measured reflections
4164 independent reflections
3442 reflections with *I* > 2σ(*I*)
*R*_int_ = 0.031

#### Refinement

*R*[*F* ^2^ > 2σ(*F* ^2^)] = 0.037
*wR*(*F* ^2^) = 0.103
*S* = 1.00
4164 reflections
239 parameters
H-atom parameters constrained
Δρ_max_ = 0.23 e Å^−3^
Δρ_min_ = −0.24 e Å^−3^

### 

Data collection: *APEX2* (Bruker, 2009 ▶); cell refinement: *SAINT* (Bruker, 2009 ▶); data reduction: *SAINT*; program(s) used to solve structure: *SHELXS97* (Sheldrick, 2008 ▶); program(s) used to refine structure: *SHELXL97* (Sheldrick, 2008 ▶); molecular graphics: *X-SEED* (Barbour, 2001 ▶); software used to prepare material for publication: *publCIF* (Westrip, 2010 ▶).

## Supplementary Material

Crystal structure: contains datablocks global, I. DOI: 10.1107/S1600536810023238/cv2733sup1.cif

Structure factors: contains datablocks I. DOI: 10.1107/S1600536810023238/cv2733Isup2.hkl

Additional supplementary materials:  crystallographic information; 3D view; checkCIF report

## Figures and Tables

**Fig. 1.**
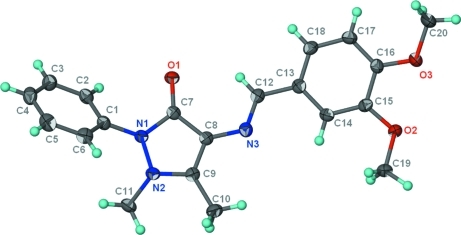
Displacement ellipsoid plot (Barbour, 2001) of C20H21N3O3 at the 70% probability level; hydrogen atoms are drawn as spheres of arbitrary radius.

## supplementary crystallographic information

### Comment

4-Aminoantipyrine (4-amino-1,2-dihydro-1,5-dimethyl-2-phenyl-3*H*-pyrazol-3-one) possesses an aminopyrazolone unit, a feature that allows the compound to condense with aromatic aldehydes to yield Schiff bases. The Schiff base derived from the benzaldehyde homolog has nearly coplanar phenyl and pyrazoly rings (Montalvo-González & Ariza-Castolo, 2003). In the title benzaldehyde analog (Scheme I, Fig. 1), the 6-membered ring is nearly coplanar with 5-membered pyrazolyl ring [dihedral angle between the two systems 11.4 (1) °]. The phenyl ring connected to the pyrazolyl ring is aligned at 45.5 (1)°.

### Experimental

3,4-Dimethoxybenzaldehyde (0.36 g, 2.2 mmol) and 4-aminoantipyrine (0.45 g, 2.2 mmol) here heated in methanol (15 ml) for 5 h to afford a colorless precipitate. The solid material was collected and recrystallized from methanol.

### Refinement

Carbon-bound H-atoms were placed in calculated positions [C–H 0.95 to 0.98 Å, *U*(H) 1.2 to 1.5*U*_eq_(C)] and were included in the refinement in the riding model approximation.

### Crystal data

**Table:**

C_20_H_21_N_3_O_3_	*F*(000) = 744
*M**_r_* = 351.40	*D*_x_ = 1.286 Mg m^−^^3^
Monoclinic, *P*2_1_/*c*	Mo *K*α radiation, λ = 0.71073 Å
Hall symbol: -P 2ybc	Cell parameters from 6153 reflections
*a* = 12.5584 (8) Å	θ = 2.4–28.2°
*b* = 10.4752 (7) Å	µ = 0.09 mm^−^^1^
*c* = 14.6002 (9) Å	*T* = 100 K
β = 109.039 (1)°	Prism, colourless
*V* = 1815.6 (2) Å^3^	0.35 × 0.25 × 0.15 mm
*Z* = 4	

### Data collection

**Table:**

Bruker SMART APEX diffractometer	3442 reflections with *I* > 2σ(*I*)
Radiation source: fine-focus sealed tube	*R*_int_ = 0.031
graphite	θ_max_ = 27.5°, θ_min_ = 1.7°
ω scans	*h* = −16→16
16900 measured reflections	*k* = −13→12
4164 independent reflections	*l* = −18→17

### Refinement

**Table:**

Refinement on *F*^2^	Primary atom site location: structure-invariant direct methods
Least-squares matrix: full	Secondary atom site location: difference Fourier map
*R*[*F*^2^ > 2σ(*F*^2^)] = 0.037	Hydrogen site location: inferred from neighbouring sites
*wR*(*F*^2^) = 0.103	H-atom parameters constrained
*S* = 1.00	*w* = 1/[σ^2^(*F*_o_^2^) + (0.0548*P*)^2^ + 0.5659*P*] where *P* = (*F*_o_^2^ + 2*F*_c_^2^)/3
4164 reflections	(Δ/σ)_max_ = 0.001
239 parameters	Δρ_max_ = 0.23 e Å^−^^3^
0 restraints	Δρ_min_ = −0.24 e Å^−^^3^

### Fractional atomic coordinates and isotropic or equivalent isotropic displacement parameters (Å^2^)

**Table:**

	*x*	*y*	*z*	*U*_iso_*/*U*_eq_	
O1	0.28425 (6)	0.77513 (8)	0.59527 (6)	0.01980 (19)	
O2	0.86240 (7)	0.62990 (8)	0.50221 (6)	0.02147 (19)	
O3	0.86452 (8)	0.84966 (8)	0.42541 (7)	0.0292 (2)	
N1	0.26678 (8)	0.60441 (9)	0.69085 (7)	0.0185 (2)	
N2	0.33485 (8)	0.50111 (9)	0.73701 (7)	0.0190 (2)	
N3	0.50498 (8)	0.62594 (9)	0.60372 (7)	0.0187 (2)	
C1	0.18777 (9)	0.65675 (11)	0.73138 (8)	0.0189 (2)	
C2	0.09077 (9)	0.71284 (11)	0.66951 (9)	0.0204 (2)	
H2	0.0778	0.7155	0.6017	0.025*	
C3	0.01286 (10)	0.76508 (12)	0.70792 (9)	0.0235 (3)	
H3	−0.0530	0.8052	0.6662	0.028*	
C4	0.03033 (10)	0.75915 (12)	0.80662 (9)	0.0252 (3)	
H4	−0.0240	0.7936	0.8323	0.030*	
C5	0.12751 (11)	0.70267 (13)	0.86781 (9)	0.0258 (3)	
H5	0.1394	0.6982	0.9354	0.031*	
C6	0.20713 (10)	0.65278 (12)	0.83089 (9)	0.0230 (3)	
H6	0.2745	0.6161	0.8731	0.028*	
C7	0.31965 (9)	0.67280 (11)	0.63587 (8)	0.0171 (2)	
C8	0.41934 (9)	0.59911 (11)	0.64197 (8)	0.0173 (2)	
C9	0.42229 (9)	0.49573 (11)	0.70009 (8)	0.0179 (2)	
C10	0.50361 (10)	0.38826 (12)	0.72347 (9)	0.0231 (3)	
H10A	0.5663	0.4077	0.6996	0.035*	
H10B	0.4657	0.3102	0.6924	0.035*	
H10C	0.5326	0.3759	0.7938	0.035*	
C11	0.27490 (10)	0.38645 (11)	0.75111 (9)	0.0225 (3)	
H11A	0.2343	0.4054	0.7965	0.034*	
H11B	0.3292	0.3177	0.7774	0.034*	
H11C	0.2212	0.3597	0.6888	0.034*	
C12	0.49724 (9)	0.72136 (11)	0.54678 (8)	0.0191 (2)	
H12	0.4304	0.7713	0.5274	0.023*	
C13	0.59014 (9)	0.75419 (11)	0.51128 (8)	0.0187 (2)	
C14	0.68242 (9)	0.67130 (11)	0.52633 (8)	0.0182 (2)	
H14	0.6838	0.5919	0.5582	0.022*	
C15	0.77102 (9)	0.70504 (11)	0.49495 (8)	0.0186 (2)	
C16	0.77122 (10)	0.82494 (11)	0.45064 (9)	0.0213 (2)	
C17	0.68007 (11)	0.90626 (12)	0.43516 (9)	0.0241 (3)	
H17	0.6794	0.9867	0.4049	0.029*	
C18	0.58924 (10)	0.86946 (11)	0.46420 (9)	0.0222 (3)	
H18	0.5257	0.9242	0.4515	0.027*	
C19	0.87115 (10)	0.51317 (11)	0.55464 (9)	0.0229 (3)	
H19A	0.9404	0.4687	0.5564	0.034*	
H19B	0.8060	0.4590	0.5226	0.034*	
H19C	0.8730	0.5316	0.6209	0.034*	
C20	0.86692 (15)	0.96944 (14)	0.37878 (14)	0.0452 (4)	
H20A	0.9381	0.9775	0.3652	0.068*	
H20B	0.8605	1.0393	0.4213	0.068*	
H20C	0.8038	0.9735	0.3179	0.068*	

### Atomic displacement parameters (Å^2^)

**Table:**

	*U*^11^	*U*^22^	*U*^33^	*U*^12^	*U*^13^	*U*^23^
O1	0.0190 (4)	0.0167 (4)	0.0230 (4)	0.0009 (3)	0.0060 (3)	0.0034 (3)
O2	0.0217 (4)	0.0182 (4)	0.0269 (4)	0.0041 (3)	0.0112 (3)	0.0044 (3)
O3	0.0333 (5)	0.0184 (4)	0.0463 (6)	0.0023 (4)	0.0273 (4)	0.0066 (4)
N1	0.0171 (4)	0.0173 (5)	0.0208 (5)	0.0008 (4)	0.0060 (4)	0.0036 (4)
N2	0.0183 (4)	0.0165 (5)	0.0216 (5)	0.0006 (4)	0.0057 (4)	0.0036 (4)
N3	0.0178 (4)	0.0192 (5)	0.0192 (5)	−0.0019 (4)	0.0062 (4)	−0.0020 (4)
C1	0.0170 (5)	0.0173 (5)	0.0228 (6)	−0.0038 (4)	0.0071 (4)	−0.0013 (5)
C2	0.0188 (5)	0.0214 (6)	0.0208 (6)	−0.0040 (4)	0.0060 (4)	−0.0001 (5)
C3	0.0176 (5)	0.0232 (6)	0.0293 (6)	−0.0018 (5)	0.0071 (5)	−0.0010 (5)
C4	0.0241 (6)	0.0237 (6)	0.0319 (7)	−0.0058 (5)	0.0147 (5)	−0.0083 (5)
C5	0.0302 (6)	0.0271 (7)	0.0212 (6)	−0.0061 (5)	0.0097 (5)	−0.0051 (5)
C6	0.0220 (6)	0.0236 (6)	0.0211 (6)	−0.0021 (5)	0.0039 (5)	−0.0013 (5)
C7	0.0164 (5)	0.0169 (5)	0.0167 (5)	−0.0032 (4)	0.0038 (4)	−0.0016 (4)
C8	0.0163 (5)	0.0172 (5)	0.0174 (5)	−0.0008 (4)	0.0042 (4)	−0.0013 (4)
C9	0.0169 (5)	0.0179 (5)	0.0170 (5)	−0.0012 (4)	0.0030 (4)	−0.0019 (4)
C10	0.0232 (6)	0.0190 (6)	0.0258 (6)	0.0036 (5)	0.0064 (5)	0.0027 (5)
C11	0.0249 (6)	0.0185 (6)	0.0251 (6)	−0.0033 (5)	0.0096 (5)	0.0031 (5)
C12	0.0184 (5)	0.0198 (6)	0.0189 (5)	0.0007 (4)	0.0060 (4)	−0.0012 (4)
C13	0.0207 (5)	0.0190 (6)	0.0171 (5)	−0.0008 (4)	0.0070 (4)	−0.0022 (4)
C14	0.0219 (5)	0.0160 (5)	0.0170 (5)	−0.0006 (4)	0.0069 (4)	0.0001 (4)
C15	0.0206 (5)	0.0161 (5)	0.0192 (5)	0.0017 (4)	0.0069 (4)	−0.0019 (4)
C16	0.0250 (6)	0.0184 (6)	0.0253 (6)	−0.0005 (5)	0.0149 (5)	−0.0008 (5)
C17	0.0325 (6)	0.0166 (6)	0.0284 (6)	0.0030 (5)	0.0169 (5)	0.0034 (5)
C18	0.0256 (6)	0.0191 (6)	0.0241 (6)	0.0046 (5)	0.0113 (5)	0.0006 (5)
C19	0.0246 (6)	0.0176 (6)	0.0273 (6)	0.0028 (5)	0.0095 (5)	0.0041 (5)
C20	0.0552 (9)	0.0222 (7)	0.0797 (12)	0.0092 (7)	0.0513 (9)	0.0175 (7)

### Geometric parameters (Å, °)

**Table:**

O1—C7	1.2354 (14)	C9—C10	1.4830 (16)
O2—C15	1.3669 (14)	C10—H10A	0.9800
O2—C19	1.4277 (14)	C10—H10B	0.9800
O3—C16	1.3627 (14)	C10—H10C	0.9800
O3—C20	1.4326 (16)	C11—H11A	0.9800
N1—C7	1.3950 (14)	C11—H11B	0.9800
N1—N2	1.4072 (13)	C11—H11C	0.9800
N1—C1	1.4206 (15)	C12—C13	1.4638 (16)
N2—C9	1.3731 (15)	C12—H12	0.9500
N2—C11	1.4673 (14)	C13—C18	1.3876 (17)
N3—C12	1.2833 (15)	C13—C14	1.4067 (16)
N3—C8	1.3926 (14)	C14—C15	1.3804 (16)
C1—C2	1.3883 (16)	C14—H14	0.9500
C1—C6	1.3933 (17)	C15—C16	1.4132 (16)
C2—C3	1.3890 (17)	C16—C17	1.3851 (17)
C2—H2	0.9500	C17—C18	1.3946 (17)
C3—C4	1.3865 (18)	C17—H17	0.9500
C3—H3	0.9500	C18—H18	0.9500
C4—C5	1.3879 (19)	C19—H19A	0.9800
C4—H4	0.9500	C19—H19B	0.9800
C5—C6	1.3839 (18)	C19—H19C	0.9800
C5—H5	0.9500	C20—H20A	0.9800
C6—H6	0.9500	C20—H20B	0.9800
C7—C8	1.4486 (15)	C20—H20C	0.9800
C8—C9	1.3688 (16)		
			
C15—O2—C19	116.78 (9)	H10B—C10—H10C	109.5
C16—O3—C20	116.62 (10)	N2—C11—H11A	109.5
C7—N1—N2	109.98 (9)	N2—C11—H11B	109.5
C7—N1—C1	124.72 (10)	H11A—C11—H11B	109.5
N2—N1—C1	119.67 (9)	N2—C11—H11C	109.5
C9—N2—N1	106.39 (9)	H11A—C11—H11C	109.5
C9—N2—C11	122.28 (10)	H11B—C11—H11C	109.5
N1—N2—C11	115.93 (9)	N3—C12—C13	120.78 (10)
C12—N3—C8	120.78 (10)	N3—C12—H12	119.6
C2—C1—C6	120.57 (11)	C13—C12—H12	119.6
C2—C1—N1	118.44 (10)	C18—C13—C14	119.19 (11)
C6—C1—N1	120.99 (10)	C18—C13—C12	120.08 (10)
C1—C2—C3	119.23 (11)	C14—C13—C12	120.71 (10)
C1—C2—H2	120.4	C15—C14—C13	120.15 (11)
C3—C2—H2	120.4	C15—C14—H14	119.9
C4—C3—C2	120.56 (11)	C13—C14—H14	119.9
C4—C3—H3	119.7	O2—C15—C14	125.03 (10)
C2—C3—H3	119.7	O2—C15—C16	114.87 (10)
C3—C4—C5	119.73 (11)	C14—C15—C16	120.10 (10)
C3—C4—H4	120.1	O3—C16—C17	125.23 (11)
C5—C4—H4	120.1	O3—C16—C15	115.00 (10)
C6—C5—C4	120.37 (12)	C17—C16—C15	119.77 (11)
C6—C5—H5	119.8	C16—C17—C18	119.66 (11)
C4—C5—H5	119.8	C16—C17—H17	120.2
C5—C6—C1	119.50 (11)	C18—C17—H17	120.2
C5—C6—H6	120.2	C13—C18—C17	121.05 (11)
C1—C6—H6	120.2	C13—C18—H18	119.5
O1—C7—N1	123.87 (10)	C17—C18—H18	119.5
O1—C7—C8	131.33 (10)	O2—C19—H19A	109.5
N1—C7—C8	104.77 (9)	O2—C19—H19B	109.5
C9—C8—N3	122.68 (10)	H19A—C19—H19B	109.5
C9—C8—C7	107.92 (10)	O2—C19—H19C	109.5
N3—C8—C7	129.26 (10)	H19A—C19—H19C	109.5
C8—C9—N2	110.34 (10)	H19B—C19—H19C	109.5
C8—C9—C10	128.36 (11)	O3—C20—H20A	109.5
N2—C9—C10	121.31 (10)	O3—C20—H20B	109.5
C9—C10—H10A	109.5	H20A—C20—H20B	109.5
C9—C10—H10B	109.5	O3—C20—H20C	109.5
H10A—C10—H10B	109.5	H20A—C20—H20C	109.5
C9—C10—H10C	109.5	H20B—C20—H20C	109.5
H10A—C10—H10C	109.5		
			
C7—N1—N2—C9	−8.00 (12)	C7—C8—C9—N2	−3.61 (13)
C1—N1—N2—C9	−162.78 (10)	N3—C8—C9—C10	−8.03 (19)
C7—N1—N2—C11	−147.65 (10)	C7—C8—C9—C10	175.83 (11)
C1—N1—N2—C11	57.57 (13)	N1—N2—C9—C8	7.08 (12)
C7—N1—C1—C2	58.12 (15)	C11—N2—C9—C8	143.54 (11)
N2—N1—C1—C2	−151.03 (10)	N1—N2—C9—C10	−172.41 (10)
C7—N1—C1—C6	−121.50 (12)	C11—N2—C9—C10	−35.94 (16)
N2—N1—C1—C6	29.34 (16)	C8—N3—C12—C13	176.26 (10)
C6—C1—C2—C3	−0.05 (17)	N3—C12—C13—C18	−168.62 (11)
N1—C1—C2—C3	−179.68 (10)	N3—C12—C13—C14	9.85 (17)
C1—C2—C3—C4	−1.31 (18)	C18—C13—C14—C15	0.35 (17)
C2—C3—C4—C5	1.22 (18)	C12—C13—C14—C15	−178.13 (10)
C3—C4—C5—C6	0.25 (19)	C19—O2—C15—C14	−6.31 (16)
C4—C5—C6—C1	−1.59 (19)	C19—O2—C15—C16	174.22 (10)
C2—C1—C6—C5	1.50 (18)	C13—C14—C15—O2	−177.20 (10)
N1—C1—C6—C5	−178.89 (11)	C13—C14—C15—C16	2.23 (17)
N2—N1—C7—O1	−172.69 (10)	C20—O3—C16—C17	−0.3 (2)
C1—N1—C7—O1	−19.46 (17)	C20—O3—C16—C15	178.96 (13)
N2—N1—C7—C8	5.75 (12)	O2—C15—C16—O3	−2.53 (15)
C1—N1—C7—C8	158.98 (10)	C14—C15—C16—O3	177.98 (11)
C12—N3—C8—C9	177.56 (11)	O2—C15—C16—C17	176.81 (11)
C12—N3—C8—C7	−7.18 (18)	C14—C15—C16—C17	−2.68 (18)
O1—C7—C8—C9	176.90 (12)	O3—C16—C17—C18	179.80 (12)
N1—C7—C8—C9	−1.37 (12)	C15—C16—C17—C18	0.52 (19)
O1—C7—C8—N3	1.1 (2)	C14—C13—C18—C17	−2.54 (18)
N1—C7—C8—N3	−177.18 (11)	C12—C13—C18—C17	175.95 (11)
N3—C8—C9—N2	172.54 (10)	C16—C17—C18—C13	2.10 (19)
